# Supplementary figures and images for: An RNA-based feed-forward mechanism ensures motor switching in oskar mRNA transport
Source: J Cell Biol. 2023 May 22;222(7):e202301113. doi: 10.1083/jcb.202301113 (PMC10202831; doi:10.1083/jcb.202301113)

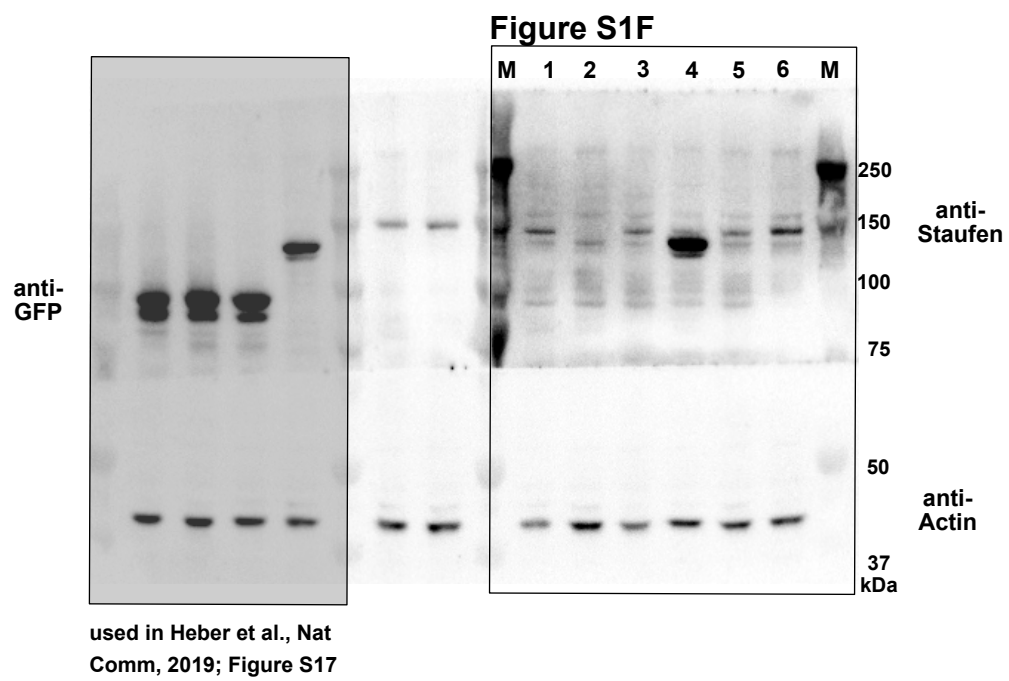

Supplement: SourceData FS1 — is the source file for Fig. S1. [file JCB_202301113_SourceDataFS1.pdf]

**Figure S4D**

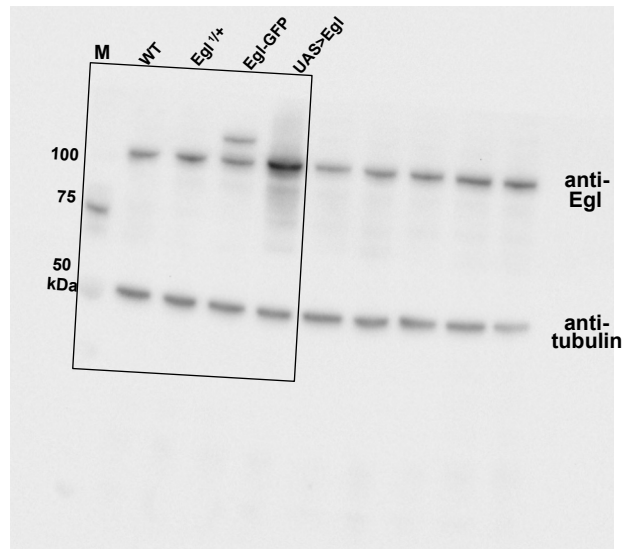

**Figure S4H**

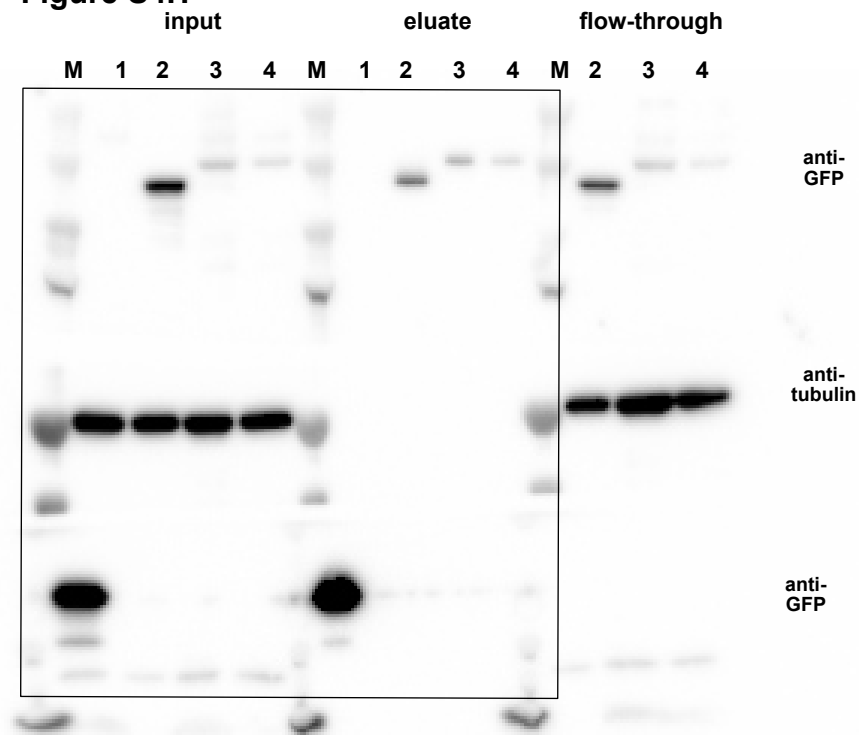

Supplement: SourceData FS4 — is the source file for Fig. S4. [file JCB_202301113_SourceDataFS4.pdf]
